# μ_2_-Oxalato-bis­[triphen­yl(thio­urea-κ*S*)tin(IV)]

**DOI:** 10.1107/S1600536812040706

**Published:** 2012-10-06

**Authors:** Yaya Sow, Libasse Diop, Kieran C. Molloy, Gabrielle Kociok-Kohn

**Affiliations:** a Laboratoire de Chimie Minerale et Analytique (LACHIMIA), Departement de Chimie, Faculte des Sciences et Techniques, Universite Cheikh, Anta Diop Dakar Senegal; b Department of Chemistry, University of Bath, Bath BA2 7AY, England

## Abstract

The asymmetric unit of the binuclear title compound, [Sn_2_(C_2_O_4_)(C_6_H_5_)_6_(CH_4_N_2_S)_2_], consists of one half of the organotin(IV) mol­ecule. The remainder is generated by a twofold rotation axis passing through the mid-point of the oxalate C—C bond. The Sn^IV^ atom exhibits a distorted trigonal–bipyramidal coordination environment with the phenyl groups in equatorial positions and the thio­urea and the monodentately bridging oxalate anion in axial positions. The mol­ecules are linked through N—H⋯O hydrogen bonds involving the amino group of the thio­urea ligand and the uncoordinating oxalate O atoms, forming layers parallel to (001). Weak C—H⋯O inter­actions are also present.

## Related literature

For background to organotin(IV) chemistry, see: Evans & Karpel (1985 ▶); Gielen *et al.* (1995 ▶). For triphenyl­tin(IV)-containing compounds and their biological activity, see: Kamruddin *et al.* (1996 ▶). For related compounds, see: Diallo *et al.* (2009 ▶); Diasse-Sarr *et al.* (1997 ▶); Diop *et al.* (1997 ▶, 1999 ▶, 2003 ▶); Tiekink (1992 ▶).
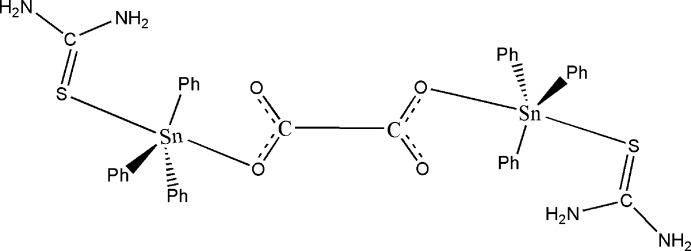


## Experimental

### 

#### Crystal data

[Sn_2_(C_2_O_4_)(C_6_H_5_)_6_(CH_4_N_2_S)_2_]
*M* *_r_* = 940.24
Monoclinic, 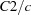

*a* = 12.9161 (2) Å
*b* = 13.9870 (2) Å
*c* = 21.8215 (3) Å
β = 99.238 (1)°
*V* = 3891.09 (10) Å^3^
*Z* = 4
Mo *K*α radiation
μ = 1.44 mm^−1^
*T* = 150 K
0.30 × 0.30 × 0.20 mm

#### Data collection

Nonius KappaCCD diffractometer
Absorption correction: multi-scan (*SORTAV*; Blessing, 1995 ▶) *T*_min_ = 0.659, *T*_max_ = 0.747
31403 measured reflections
4472 independent reflections
3665 reflections with *I* > 2σ(*I*)
*R*_int_ = 0.057

#### Refinement

*R*[*F* ^2^ > 2σ(*F* ^2^)] = 0.030
*wR*(*F* ^2^) = 0.066
*S* = 1.09
4472 reflections
251 parameters
H atoms treated by a mixture of independent and constrained refinement
Δρ_max_ = 1.34 e Å^−3^
Δρ_min_ = −1.11 e Å^−3^

### 

Data collection: *COLLECT* (Nonius, 1999 ▶); cell refinement: *DENZO* and *SCALEPACK* (Otwinowski & Minor, 1997 ▶); data reduction: *DENZO* and *SCALEPACK*; program(s) used to solve structure: *SIR97* (Altomare *et al.*, 1999 ▶); program(s) used to refine structure: *SHELXL97* (Sheldrick, 2008 ▶); molecular graphics: *ORTEP-3* (Farrugia, 1997 ▶); software used to prepare material for publication: *WinGX* (Farrugia,1999 ▶).

## Supplementary Material

Crystal structure: contains datablock(s) I, New_Global_Publ_Block. DOI: 10.1107/S1600536812040706/wm2662sup1.cif

Structure factors: contains datablock(s) I. DOI: 10.1107/S1600536812040706/wm2662Isup2.hkl

Additional supplementary materials:  crystallographic information; 3D view; checkCIF report

## Figures and Tables

**Table 1:** Hydrogen-bond geometry (Å, °)

*D*—H⋯*A*	*D*—H	H⋯*A*	*D*⋯*A*	*D*—H⋯*A*
N1—H1*A*⋯O2^i^	0.81 (4)	2.06 (4)	2.824 (3)	157 (4)
N2—H2*A*⋯O2^ii^	0.86 (4)	2.14 (4)	2.970 (3)	164 (3)
C6—H6⋯O1	0.95	2.44	2.957 (3)	114
C18—H18⋯O2	0.95	2.39	3.234 (3)	147

Symmetry codes: (i) 
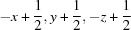
; (ii) 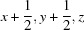.

**Fig. 1.**
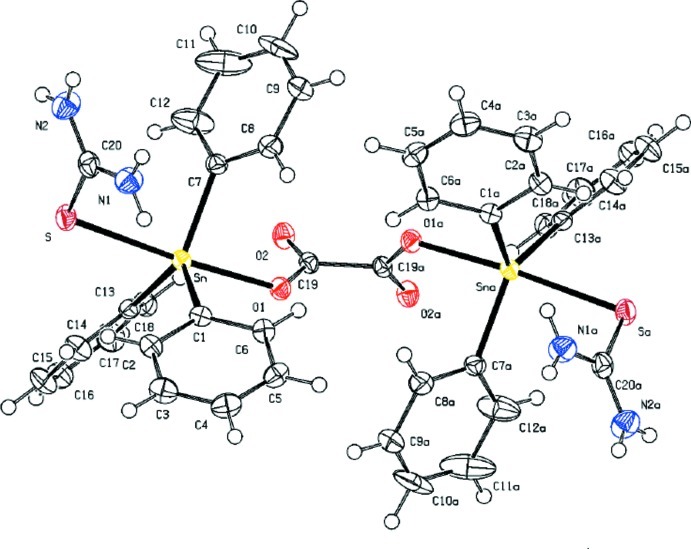
The molecule of the title complex showing the numbering scheme. Displacement ellipsoids are drawn at the 50% probability level. [Symmetry code a) -x, y, -z + 1/2.]

**Fig. 2.**
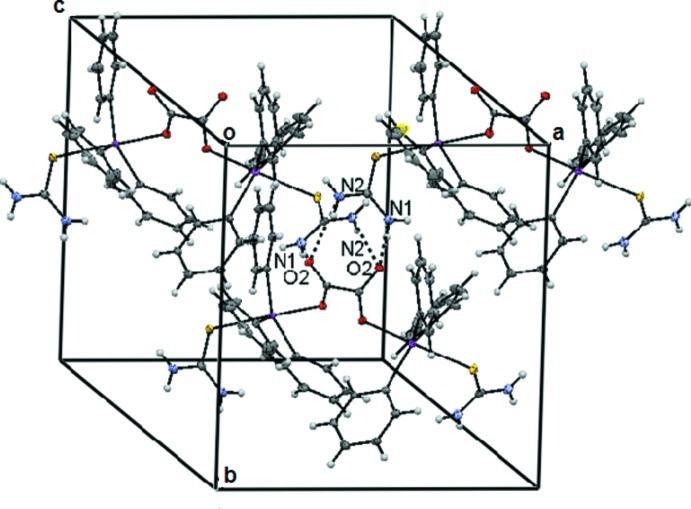
View of the N—H···O hydrogen bonding system (dashed lines) assured by pairs of oxygen atoms of the oxalate and H atoms of thiourea.

**Fig. 3.**
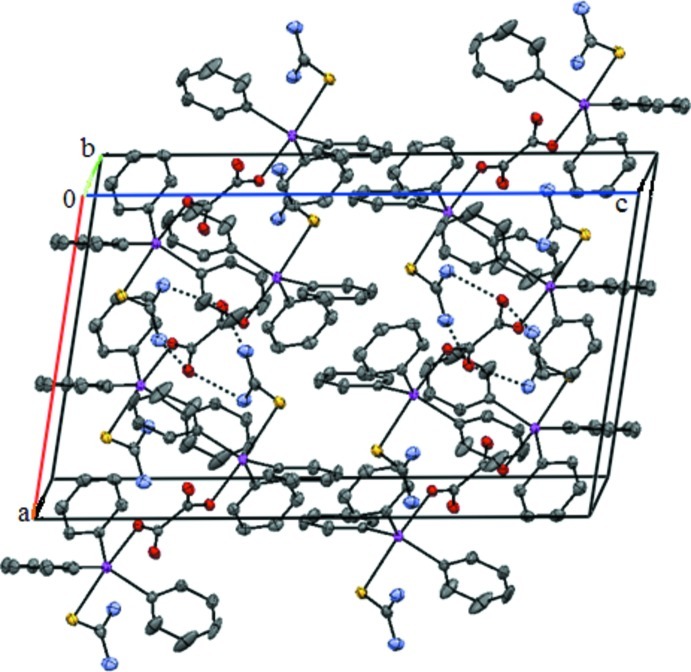
The packing of the structure showing N—H···O hydrogen bonding interactions as dashed lines

## supplementary crystallographic information

### Comment

Interest in organotin (IV) chemistry remains high because of several applications found in molecules belonging to this family (Evans & Karpel, 1985; Gielen *et al.*, 1995), specifically triphenyltin(IV)-residue containing compounds for which biological activity has been reported (Kamruddin *et al.*, 1996). Our specific interest lies also in the coordinating behavior of oxy-anions to these organometallic centers; we have previously published several crystal structures dealing with such systems (Diallo *et al.*, 2009; Diasse-Sarr *et al.*, 1997; Diop *et al.*, 1997). Moreover, we have reported spectroscopic data (Diop *et al.*, 1999) and the crystal structure of [Sn_2_(C_2_O_4_)(C_6_H_5_)_6_] which exhibits tetrahedrally coordinate tin atoms (Diop *et al.*, 2003). Here we report a study of the interactions between this species and thiourea, which has yielded the title compound, [Sn_2_(C_2_O_4_)(C_6_H_5_)_6_(CH_4_N_2_S)_2_].

The molecule of the title compound has site symmetry 2 with the twofold rotation axcis passing through the mid-point of the central oxalate C—C bond. The Sn(IV) atom is five-coordinate by one oxygen atom of the oxalate anion, a sulfur atom of the thiourea ligand [Sn—O 2.2471 (17), Sn—S 2.6945 (7) Å] which are in apical positions and to three phenyl groups [Sn—C 2.146 (2), 2.139 (2), 2.139 (2) Å] occupying the equatorial positions of the trigonal bipyramid (Fig. 1). The Sn—S bond length is longer than the Sn—S bond length [2.573 (1) Å] found, for example, in {*^t^*(C_4_H_9_)_2_Sn[S_2_CN(CH_3_)_2_]_2_} which contains a trigonal bipyramidally coordinate tin(IV) atom (Tiekink, 1992). The angle S—Sn—O [175.97 (5)°] deviates slightly from linearity. The sum of the C—Sn—C angles (359.96°) indicates a nearly perfectly planar Sn(C_6_H_5_)_3_ residue consistent with the near linearity of the axial substituents. The Sn—O bond length is remarkably long when compared with the Sn—O distance [2.111 (1) Å] in the tetrahedrally coordinate tin(IV) atom in [Sn_2_(C_2_O_4_)(C_6_H_5_)_6_] (Diop *et al.*, 2003). The addition of SC(NH_2_)_2_ apparently has caused a change in the coordination from tetrahedral to trigonal-bipyramidal along with a Sn—O bond length increase. The two C—O bond length of the oxalate anion are slightly different because the O atom of the C19—O1 bond [1.269 (3) Å] is also involved in bonding to the Sn(IV) atom, whereas the O atom of the C19—O2 bond [1.243 (3) Å] is involved in hydrogen bonding with the amino group. These interactions lead to the formation of layers parallel to (001) (Figs. 2,3). Weak C—H···O hydrogen bonding is also observed.

### Experimental

All chemicals were purchased from Aldrich or Merck and used without any further purification. [Sn_2_(C_2_O_4_)(C_6_H_5_)_6_] has been obtained on allowing Sn(C_6_H_5_)_3_OH to react with oxalic acid in a 2:1 ratio in ethanol. A white powder is collected after slow evaporation. When [Sn_2_(C_2_O_4_)(C_6_H_5_)_6_] is mixed with SC(NH_2_)_2_ in a 1:2 ratio, both as ethanolic solutions, a colorless solution is obtained which gives crystals of [Sn_2_(C_2_O_4_)(C_6_H_5_)_6_(CH_4_N_2_S)_2_] suitable for X-ray work, after a slow solvent evaporation.

### Refinement

The maximum remaining electron density is 0.79 Å from C3 while the minimum density is in the immediate vicinity of tin. Hydrogen atoms bonded to the N atom have been located in difference Fourier maps and have been freely refined. The other hydrogen atoms have been placed onto calculated position and refined using a riding model, with C—H distances of 0.95 Å and *U*_iso_(H)= 1.2*U*_eq_(C).

### Crystal data

**Table:**

[Sn_2_(C_2_O_4_)(C_6_H_5_)_6_(CH_4_N_2_S)_2_]	*F*(000) = 1880
*M**_r_* = 940.24	*D*_x_ = 1.605 Mg m^−^^3^
Monoclinic, *C*2/*c*	Mo *K*α radiation, λ = 0.71073 Å
Hall symbol: -C 2yc	Cell parameters from 26977 reflections
*a* = 12.9161 (2) Å	θ = 2.9–27.5°
*b* = 13.9870 (2) Å	µ = 1.44 mm^−^^1^
*c* = 21.8215 (3) Å	*T* = 150 K
β = 99.238 (1)°	Block, colourless
*V* = 3891.09 (10) Å^3^	0.30 × 0.30 × 0.20 mm
*Z* = 4	

### Data collection

**Table:**

Nonius KappaCCD diffractometer	4472 independent reflections
Radiation source: fine-focus sealed tube	3665 reflections with *I* > 2σ(*I*)
Graphite monochromator	*R*_int_ = 0.057
400 1.5 degree images with φ and ω scans	θ_max_ = 27.5°, θ_min_ = 3.8°
Absorption correction: multi-scan (*SORTAV*; Blessing, 1995)	*h* = −16→16
*T*_min_ = 0.659, *T*_max_ = 0.747	*k* = −18→18
31403 measured reflections	*l* = −28→28

### Refinement

**Table:**

Refinement on *F*^2^	Primary atom site location: structure-invariant direct methods
Least-squares matrix: full	Secondary atom site location: difference Fourier map
*R*[*F*^2^ > 2σ(*F*^2^)] = 0.030	Hydrogen site location: inferred from neighbouring sites
*wR*(*F*^2^) = 0.066	H atoms treated by a mixture of independent and constrained refinement
*S* = 1.09	*w* = 1/[σ^2^(*F*_o_^2^) + (0.0248*P*)^2^ + 7.4801*P*] where *P* = (*F*_o_^2^ + 2*F*_c_^2^)/3
4472 reflections	(Δ/σ)_max_ = 0.001
251 parameters	Δρ_max_ = 1.34 e Å^−^^3^
0 restraints	Δρ_min_ = −1.11 e Å^−^^3^

### Special details

**Table:**

Geometry. All e.s.d.'s (except the e.s.d. in the dihedral angle between two l.s. planes) are estimated using the full covariance matrix. The cell e.s.d.'s are taken into account individually in the estimation of e.s.d.'s in distances, angles and torsion angles; correlations between e.s.d.'s in cell parameters are only used when they are defined by crystal symmetry. An approximate (isotropic) treatment of cell e.s.d.'s is used for estimating e.s.d.'s involving l.s. planes.
Refinement. Refinement of *F*^2^ against ALL reflections. The weighted *R*-factor *wR* and goodness of fit *S* are based on *F*^2^, conventional *R*-factors *R* are based on *F*, with *F* set to zero for negative *F*^2^. The threshold expression of *F*^2^ > σ(*F*^2^) is used only for calculating *R*-factors(gt) *etc*. and is not relevant to the choice of reflections for refinement. *R*-factors based on *F*^2^ are statistically about twice as large as those based on *F*, and *R*- factors based on ALL data will be even larger.

### Fractional atomic coordinates and isotropic or equivalent isotropic displacement parameters (Å^2^)

**Table:**

	*x*	*y*	*z*	*U*_iso_*/*U*_eq_	
Sn	0.165377 (13)	0.128741 (12)	0.141781 (7)	0.01783 (7)	
S	0.33347 (5)	0.18709 (5)	0.09290 (3)	0.02648 (16)	
O1	0.03210 (13)	0.08111 (13)	0.18919 (8)	0.0223 (4)	
O2	0.09855 (14)	−0.05868 (13)	0.22806 (8)	0.0247 (4)	
N1	0.3214 (2)	0.34372 (19)	0.16116 (13)	0.0319 (6)	
H1A	0.340 (3)	0.385 (3)	0.1866 (18)	0.050 (12)*	
H1B	0.256 (3)	0.351 (2)	0.1449 (16)	0.040 (10)*	
N2	0.4815 (2)	0.2725 (2)	0.17068 (13)	0.0342 (6)	
H2A	0.509 (3)	0.319 (3)	0.1931 (16)	0.044 (10)*	
H2B	0.519 (3)	0.228 (3)	0.1620 (17)	0.050 (12)*	
C1	0.07308 (19)	0.25438 (17)	0.11628 (11)	0.0190 (5)	
C2	0.0815 (2)	0.30357 (19)	0.06162 (12)	0.0245 (6)	
H2	0.1363	0.2876	0.0392	0.029*	
C3	0.0110 (2)	0.3755 (2)	0.03950 (14)	0.0308 (6)	
H3	0.0181	0.4082	0.0023	0.037*	
C4	−0.0697 (2)	0.3998 (2)	0.07159 (14)	0.0317 (7)	
H4	−0.1187	0.4480	0.0560	0.038*	
C5	−0.0780 (2)	0.35315 (19)	0.12646 (13)	0.0266 (6)	
H5	−0.1323	0.3702	0.1491	0.032*	
C6	−0.0075 (2)	0.28136 (19)	0.14865 (12)	0.0229 (6)	
H6	−0.0140	0.2500	0.1865	0.027*	
C7	0.2578 (2)	0.11799 (18)	0.23207 (12)	0.0215 (5)	
C8	0.2292 (2)	0.1691 (3)	0.28081 (13)	0.0410 (8)	
H8	0.1752	0.2157	0.2724	0.049*	
C9	0.2764 (3)	0.1547 (4)	0.34114 (15)	0.0574 (11)	
H9	0.2549	0.1913	0.3735	0.069*	
C10	0.3516 (3)	0.0900 (3)	0.35449 (15)	0.0576 (12)	
H10	0.3830	0.0797	0.3964	0.069*	
C11	0.3848 (4)	0.0368 (3)	0.3069 (2)	0.0700 (14)	
H11	0.4388	−0.0095	0.3162	0.084*	
C12	0.3373 (3)	0.0529 (2)	0.24521 (16)	0.0515 (10)	
H12	0.3605	0.0184	0.2125	0.062*	
C13	0.15172 (19)	0.01853 (18)	0.07300 (11)	0.0202 (5)	
C14	0.1501 (2)	0.0447 (2)	0.01093 (12)	0.0298 (6)	
H14	0.1558	0.1102	0.0004	0.036*	
C15	0.1403 (2)	−0.0245 (2)	−0.03532 (13)	0.0360 (7)	
H15	0.1396	−0.0062	−0.0773	0.043*	
C16	0.1317 (2)	−0.1194 (2)	−0.02054 (14)	0.0333 (7)	
H16	0.1249	−0.1665	−0.0523	0.040*	
C17	0.1328 (2)	−0.1461 (2)	0.04042 (14)	0.0343 (7)	
H17	0.1266	−0.2117	0.0506	0.041*	
C18	0.1429 (2)	−0.0774 (2)	0.08685 (12)	0.0270 (6)	
H18	0.1438	−0.0964	0.1287	0.032*	
C19	0.03827 (19)	0.01088 (18)	0.22640 (11)	0.0190 (5)	
C20	0.3813 (2)	0.2744 (2)	0.14526 (12)	0.0260 (6)	

### Atomic displacement parameters (Å^2^)

**Table:**

	*U*^11^	*U*^22^	*U*^33^	*U*^12^	*U*^13^	*U*^23^
Sn	0.02036 (10)	0.01769 (10)	0.01559 (9)	0.00087 (7)	0.00337 (6)	−0.00049 (7)
S	0.0272 (3)	0.0299 (4)	0.0242 (3)	−0.0054 (3)	0.0098 (3)	−0.0070 (3)
O1	0.0227 (9)	0.0238 (10)	0.0211 (9)	0.0002 (8)	0.0056 (7)	0.0060 (8)
O2	0.0294 (10)	0.0208 (10)	0.0254 (10)	0.0042 (8)	0.0093 (8)	0.0000 (8)
N1	0.0311 (15)	0.0292 (14)	0.0346 (14)	−0.0033 (12)	0.0029 (11)	−0.0081 (12)
N2	0.0297 (14)	0.0301 (15)	0.0427 (16)	−0.0074 (13)	0.0057 (12)	−0.0087 (13)
C1	0.0217 (13)	0.0153 (12)	0.0193 (12)	−0.0001 (10)	0.0011 (10)	0.0000 (10)
C2	0.0277 (14)	0.0250 (14)	0.0208 (13)	0.0001 (11)	0.0039 (11)	0.0013 (11)
C3	0.0364 (16)	0.0237 (15)	0.0309 (15)	0.0021 (13)	0.0015 (12)	0.0069 (12)
C4	0.0295 (15)	0.0247 (15)	0.0389 (17)	0.0063 (12)	−0.0006 (12)	0.0012 (13)
C5	0.0222 (14)	0.0216 (14)	0.0358 (15)	0.0014 (11)	0.0046 (11)	−0.0065 (12)
C6	0.0255 (14)	0.0197 (13)	0.0240 (13)	−0.0012 (11)	0.0054 (10)	−0.0013 (11)
C7	0.0205 (12)	0.0232 (14)	0.0201 (12)	−0.0027 (11)	0.0015 (10)	0.0026 (11)
C8	0.0254 (15)	0.072 (2)	0.0251 (15)	0.0055 (16)	0.0021 (12)	−0.0117 (15)
C9	0.0331 (18)	0.117 (4)	0.0207 (16)	−0.010 (2)	0.0009 (13)	−0.0065 (19)
C10	0.069 (3)	0.075 (3)	0.0207 (16)	−0.046 (2)	−0.0189 (16)	0.0201 (17)
C11	0.077 (3)	0.037 (2)	0.077 (3)	0.012 (2)	−0.045 (2)	0.006 (2)
C12	0.062 (2)	0.039 (2)	0.044 (2)	0.0230 (18)	−0.0196 (17)	−0.0121 (16)
C13	0.0170 (12)	0.0228 (14)	0.0202 (12)	0.0005 (10)	0.0017 (10)	−0.0059 (10)
C14	0.0387 (17)	0.0275 (15)	0.0224 (14)	0.0005 (13)	0.0024 (12)	−0.0017 (11)
C15	0.0424 (18)	0.046 (2)	0.0187 (14)	−0.0021 (15)	0.0026 (12)	−0.0073 (13)
C16	0.0330 (16)	0.0374 (18)	0.0292 (15)	−0.0032 (14)	0.0036 (12)	−0.0176 (13)
C17	0.0414 (17)	0.0265 (16)	0.0350 (16)	−0.0030 (13)	0.0064 (13)	−0.0084 (13)
C18	0.0292 (14)	0.0288 (15)	0.0234 (14)	−0.0015 (12)	0.0051 (11)	−0.0032 (12)
C19	0.0217 (13)	0.0192 (13)	0.0164 (12)	−0.0032 (11)	0.0042 (10)	−0.0016 (10)
C20	0.0303 (15)	0.0252 (14)	0.0241 (14)	−0.0062 (12)	0.0095 (11)	0.0012 (11)

### Geometric parameters (Å, º)

**Table:**

Sn—C7	2.139 (2)	C6—H6	0.9500
Sn—C13	2.139 (2)	C7—C12	1.368 (4)
Sn—C1	2.146 (2)	C7—C8	1.381 (4)
Sn—O1	2.2471 (17)	C8—C9	1.374 (4)
Sn—S	2.6945 (7)	C8—H8	0.9500
S—C20	1.718 (3)	C9—C10	1.325 (6)
O1—C19	1.269 (3)	C9—H9	0.9500
O2—C19	1.243 (3)	C10—C11	1.398 (6)
N1—C20	1.321 (4)	C10—H10	0.9500
N1—H1A	0.81 (4)	C11—C12	1.406 (5)
N1—H1B	0.86 (4)	C11—H11	0.9500
N2—C20	1.324 (4)	C12—H12	0.9500
N2—H2A	0.85 (4)	C13—C18	1.384 (4)
N2—H2B	0.82 (4)	C13—C14	1.400 (4)
C1—C2	1.396 (3)	C14—C15	1.389 (4)
C1—C6	1.400 (4)	C14—H14	0.9500
C2—C3	1.391 (4)	C15—C16	1.375 (4)
C2—H2	0.9500	C15—H15	0.9500
C3—C4	1.388 (4)	C16—C17	1.380 (4)
C3—H3	0.9500	C16—H16	0.9500
C4—C5	1.383 (4)	C17—C18	1.388 (4)
C4—H4	0.9500	C17—H17	0.9500
C5—C6	1.390 (4)	C18—H18	0.9500
C5—H5	0.9500	C19—C19^i^	1.538 (5)
			
C7—Sn—C13	124.52 (10)	C9—C8—C7	122.0 (3)
C7—Sn—C1	120.08 (9)	C9—C8—H8	119.0
C13—Sn—C1	115.36 (9)	C7—C8—H8	119.0
C7—Sn—O1	84.87 (8)	C10—C9—C8	120.5 (4)
C13—Sn—O1	97.26 (8)	C10—C9—H9	119.7
C1—Sn—O1	85.84 (8)	C8—C9—H9	119.7
C7—Sn—S	91.14 (7)	C9—C10—C11	120.1 (3)
C13—Sn—S	85.49 (7)	C9—C10—H10	120.0
C1—Sn—S	95.66 (7)	C11—C10—H10	120.0
O1—Sn—S	175.97 (5)	C10—C11—C12	119.2 (3)
C20—S—Sn	100.29 (9)	C10—C11—H11	120.4
C19—O1—Sn	123.37 (16)	C12—C11—H11	120.4
C20—N1—H1A	126 (3)	C7—C12—C11	120.3 (3)
C20—N1—H1B	123 (2)	C7—C12—H12	119.8
H1A—N1—H1B	111 (3)	C11—C12—H12	119.8
C20—N2—H2A	121 (2)	C18—C13—C14	118.4 (2)
C20—N2—H2B	119 (3)	C18—C13—Sn	123.08 (19)
H2A—N2—H2B	120 (3)	C14—C13—Sn	118.5 (2)
C2—C1—C6	117.7 (2)	C15—C14—C13	120.4 (3)
C2—C1—Sn	120.59 (19)	C15—C14—H14	119.8
C6—C1—Sn	121.12 (18)	C13—C14—H14	119.8
C3—C2—C1	121.1 (3)	C16—C15—C14	120.2 (3)
C3—C2—H2	119.4	C16—C15—H15	119.9
C1—C2—H2	119.4	C14—C15—H15	119.9
C4—C3—C2	120.3 (3)	C15—C16—C17	120.0 (3)
C4—C3—H3	119.8	C15—C16—H16	120.0
C2—C3—H3	119.8	C17—C16—H16	120.0
C5—C4—C3	119.4 (3)	C16—C17—C18	120.1 (3)
C5—C4—H4	120.3	C16—C17—H17	120.0
C3—C4—H4	120.3	C18—C17—H17	120.0
C4—C5—C6	120.3 (3)	C13—C18—C17	120.9 (3)
C4—C5—H5	119.9	C13—C18—H18	119.5
C6—C5—H5	119.9	C17—C18—H18	119.5
C5—C6—C1	121.2 (3)	O2—C19—O1	126.8 (2)
C5—C6—H6	119.4	O2—C19—C19^i^	116.57 (17)
C1—C6—H6	119.4	O1—C19—C19^i^	116.58 (18)
C12—C7—C8	117.9 (3)	N1—C20—N2	118.6 (3)
C12—C7—Sn	121.9 (2)	N1—C20—S	122.2 (2)
C8—C7—Sn	119.6 (2)	N2—C20—S	119.2 (2)

Symmetry code: (i) −*x*, *y*, −*z*+1/2.

### Hydrogen-bond geometry (Å, º)

**Table:**

*D*—H···*A*	*D*—H	H···*A*	*D*···*A*	*D*—H···*A*
N1—H1*A*···O2^ii^	0.81 (4)	2.06 (4)	2.824 (3)	157 (4)
N2—H2*A*···O2^iii^	0.86 (4)	2.14 (4)	2.970 (3)	164 (3)
C6—H6···O1	0.95	2.44	2.957 (3)	114
C18—H18···O2	0.95	2.39	3.234 (3)	147

Symmetry codes: (ii) −*x*+1/2, *y*+1/2, −*z*+1/2; (iii) *x*+1/2, *y*+1/2, *z*.
